# 

**Published:** 1915-12-25

**Authors:** 


### CONTENTS.

| | PAGE |
|---|---|
| **Hospital Administration in War-Time ... ...** | 269 |
| **The Medical Examination of Recruits ... ...** | 270 |
| **Hospital and Institutional News ... ... ... ...** | 271 |
| Visit of the Queen to Fulham Military Hospital—King Edward VII.'s Hospital Fund—The Metropolitan Hospital Sunday Fund—The League of Mercy—The Saturday Fund and the Hospital Situation—The Red Cross Budget—Bestowal of Red Cross Orders—Grumblings in the Public Health Service—Christmas in the Wards—The Wounded and the Book Market—V.A.D. Gathering at Leicester—The Case of our Merchant Seamen—Civilian Doctors and the R.A.M.C.—An Additional Instance—The Advisory Board for Medical Services—Large Reversions for Incurables' Hospitals—This Week's Drug Market. | |
| **A Reminiscence of 1914 ... ... ... ...** | 275 |
| Christmas Morning with an Ambulance. | |
| **A Talk About Voluntary Hospitals ... ...** | 277 |
| By the Rt. Hon. Lord Sandhurst, G.C.I.E., G.C.S.I. (Lord Chamberlain). | |
| **King Edward's Hospital Fund for London ...** | 279 |
| Meeting of the Governors and General Council. | |
| The Distribution Committee's Report. | |
| Grants to Hospitals Recommended by the Distribution Committee. | |
| The Convalescent Homes Committee's Report | |
| Grants Recommended by the Convalescent Homes Committee. | |
| **Metropolitan Hospital Sunday Fund ... ...** | 283 |
| Annual Meeting of Constituents. | |
| **Sir Ernest Cassel's Further Gifts to Hospitals** | 284 |
| **The League of Mercy... ... ... ... ...** | 285 |
| Annual Meeting at St. James's Palace. | |
| **Gifts to Hospitals ... ... ... ... ...** | 286 |
| **Our Voluntary Hospitals and Institutions ...** | 287 |
| What should they Publish about their Work and Needs? | |
| **The Institutional Worker ... ... (Suppl.)** | 1 |
| Institution Laundries. | |
| Question for December. (Suppl.) | 2 |
| Enquiries and Answers. | |
| Employment and Training. | |

